# Retraction Note: Overexpression of microRNA-195-5p reduces cisplatin resistance and angiogenesis in ovarian cancer by inhibiting the PSAT1-dependent GSK3β/β-catenin signaling pathway

**DOI:** 10.1186/s12967-022-03560-y

**Published:** 2022-08-04

**Authors:** Jun Dai, Rujia Wei, Peihai Zhang

**Affiliations:** 1 Department of Gynecology and Obstetrics, Qilu Hospital of Shandong University, No. 107, Wenhua West Road, Jinan, 250012 Shandong People’s Republic of China; 2 School of Life Sciences, Liaocheng University, Liaocheng, 252000 People’s Republic of China; 3 Department of Gynecology and Obstetrics, Qilu Hospital of Shandong University (Qingdao Hospital District), No. 758, Hefei Road, Shibei District, Qingdao, 266035 Shandong People’s Republic of China

## Retraction Note: J Transl Med (2019) 17:190 https://doi.org/10.1186/s12967-019-1932-1

The Editor-in-Chief has retracted this article because authors were unable to provide evidence for ethics approval prior to commencing this study.

In addition, co-author Beihua Kong stated that his name was added to the article without his knowledge and that he was neither aware of any contents of the article, nor the submission process. He also stated the email address provided on submission was not his email address, and that he did not sign any authorship form submitted to the journal.

Author Beihua Kong agrees with this retraction. Author Peihai Zhang stated on behalf of remaining co-authors that they do not agree to this retraction.

